# Efficacy and Safety of ^125^I Seed Implantation in the Treatment of Pelvic Recurrent Cervical Cancer Following Radiotherapy: A Single‐Arm Meta‐Analysis of Chinese Patients

**DOI:** 10.1002/cnr2.2147

**Published:** 2024-08-19

**Authors:** Yunxin Wang, Yuhong Ma, Lijuan Zou, Hongwei Lei, Yun Teng, Fuxiu Ye, Feng Zhang, Haichen Zhang

**Affiliations:** ^1^ Department of Radiation Oncology The Second Affiliated Hospital of Dalian Medical University Dalian China

**Keywords:** ^125^I, brachytherapy, meta‐analysis, radiotherapy, recurrent cervical cancer

## Abstract

**Background:**

The study aimed to assess the efficacy and safety of ^125^I seed implantation in the treatment of pelvic recurrent cervical cancer following radiotherapy. This meta‐analysis was registered in PROSPERO. We looked up relevant studies in the databases of CNKI, Wanfang, CBM, PubMed, Embase, Cochrane Library, and Web of Science. The endpoint measures include the objective response rate, disease control rate, progression‐free survival, overall survival, and adverse events.

**Recent Fidings:**

The meta‐analysis included six studies and a total of 246 patients. The pooled ORR of tumor response was 63%, and the DCR was 87%. The median PFS was 9.09 months, and the median OS was 13.46 months. The incidence of adverse events of Grade ≥III was 6%.

**Conclusion:**

In conclusion, this meta‐analysis confirmed that ^125^I seed implantation has a good local control rate and high safety in the treatment of pelvic recurrent cervical cancer following radiotherapy, and can be used as a remedial treatment for pelvic recurrent cervical cancer following radiotherapy to prolong the survival time of patients.

**Trial Registration:**

PROSPERO: CRD42023423857

## 1 Introduction

Cervical cancer is the fourth most common malignant tumor among women. In 2018, there were 311 000 deaths and 570 000 new cases worldwide [1]. Among them, China is the country with the highest number of new cases [1].

Despite the implementation of human papillomavirus vaccination and cervical cancer screening, the incidence and mortality rates of cervical cancer are still on the rise. At the moment, the clinical treatment of cervical cancer is, in principle, mainly surgical treatment in the early stage, and radiotherapy, supplemented by chemotherapy, in the middle and late stages. A total of 20%–40% of cervical cancer patients who undergo standard radical surgical treatment or radiotherapy will still have local recurrence or distant metastasis [2]. As for cervical cancer patients with pelvic recurrence following radiotherapy, because of the decreased radiation tolerance of normal tissues such as the small intestine, rectum, bladder, and so on, it is not suitable to perform extensive radiotherapy again, and it is difficult to achieve an effective dose of pelvic retreatment radiotherapy.

Recurrent cervical cancer is defined as cervical cancer that recurs with the same pathological type after it has been cured by the first radical surgery and radiotherapy. The cisplatin‐based combination regimen is the first‐line treatment for recurrent cervical cancer. However, disease progression can occur within 9 months after most patients receive first‐line treatment with the above chemotherapy regimens [3, 4]. Therefore, how to improve the therapeutic effect and prolong the survival of patients with recurrent cervical cancer is one of the urgent clinical challenges.

^125^I seed implantation is defined as the implantation of ^125^I seeds into tumors or tissues that may be invaded by tumors by minimally invasive (percutaneous puncture) or surgery. It can also be implanted into metastatic lymph nodes. ^125^I seeds have the following characteristics [5]: (1) The diameter is 0.8 mm, the length is 4.5 mm, and the volume is small and easy to use. (2) The half‐life period is 59.6 days, and the main emission energy is x‐ray of 27.4 and 31.4 KeV, and γ‐ray of 35.5KeV, respectively. ^125^I seeds are mainly used to inhibit the mitosis of tumor cells by releasing γ‐rays, cleaving the DNA molecular strands, thus causing tumor cells to lose the ability to proliferate [6]. At the same time, γ‐rays can also ionize water molecules in tumor cells to create oxygen free radicals, which interact with cellular nucleic acids and proteins to destroy tissue and cells [6]. Additionally, γ‐rays can destroy the expression of VEGFR, reduce the tumor's blood supply, and subsequently stop the growth of the tumor [6].

Conventional radiotherapy is defined as the radiation effect of external γ‐rays, proton rays, and electron rays on the human body. The intensity of its action depends on the size of the dose absorbed by the body. Different irradiation amounts and modes of action produce different effects. Compared with conventional external radiotherapy [7], ^125^I seed brachytherapy has several advantages. It can not only significantly increase the therapeutic dose of tumor tissue, to improve local control rate, but also effectively reduce the irradiated dose of nearby normal tissue and avoid serious complications, making it an ideal local treatment method.

Radioactive seed implantation was first used in the treatment of prostate cancer in the early 20th century, and it is the first choice for the treatment of early low‐risk prostate cancer [8]. In recent years, studies have shown that ^125^I seed implantation can improve the tumor response and survival of solid tumors such as glioma [9], head and neck cancer [10, 11], lung cancer [12], liver tumor [13, 14], and pancreatic cancer [15]. Some studies have also shown that ^125^I seed implantation is effective and safe in the treatment of pelvic recurrent cervical cancer following radiotherapy [16, 17, 18, 19, 20, 21]. Therefore, this meta‐analysis was conducted to assess the efficacy and safety of ^125^I seed implantation in the treatment of pelvic recurrent cervical cancer after radiotherapy, as well as to propose a treatment option for clinical application.

## 2 Materials and Methods

### 2.1 Search Strategy

We searched the databases, such as CNKI, Wan fang, CBM, PubMed, Embase, Cochrane Library, and Web of Science. Retrieval from the establishment of the database to June 2023. Two Chinese search terms are “cervical tumor, cervical cancer” and “^125^I, iodine‐125, particle.” The English search terms include “Uterine Cervical Neoplasms” and “^125^I radioisotope.” Subject headings and free words are searched at the same time.

### 2.2 Selection Criteria

This meta‐analysis contains studies that met the inclusion criteria listed below: (1) population: patients with recurrent pelvic cervical cancer have received radiotherapy in the past; (2) intervention: the patients only received ^125^I seed implantation; and (3) outcomes: studies documented interesting clinical tumor endpoints, including objective response rate (ORR), disease control rate (DCR), progression‐free survival (PFS), overall survival (OS), and adverse events (AEs). RECIST 1.1 [22] was used to evaluate the tumor response. CTCAE [23] was used to evaluate the incidence and severity of adverse events. The criteria for excluding the study are as follows: (1) reviews, case reports, cell or animal experiments, and letters were excluded; (2) studies with fewer than 10 patients were excluded; and (3) studies from which meaningful data could not be collected were excluded. Two independent investigators used inclusion and exclusion criteria to identify potential eligible publications. Any disagreements over study inclusion were addressed between these two or with a third investigator.

### 2.3 Data Extraction

The extracted characteristics from the included studies were summarized as follows: author, publication year, country, sample size, histology, median age, median follow‐up, past treatment regimens, and reported endpoints. Clinical and safety endpoints were measured using indexes such as ORR, DCR, OS, PFS, the incidence of any AEs, and Grade ≥III AEs. The details of each included study are described in Table 1.

**TABLE 1:** Characteristics of the studies included in the meta‐analysis.

Study, year	Country	Sample size	Histology			Mean ages, years	Median follow‐up, months	Intervention	Prior therapy	Endpoints
			SCC	ADCC	Others					
Liqiu Ji, 2021 [16]	China	21	18	3	0	49 (28–74)	13 (3.5–31)	SI	RT	ORR, DCR, PFS, OS, AEs
Shuqin Chen, 2020 [17]	China	36	32	4	0	44 (30–79)	NR	SI	RT	ORR, DCR, AEs
Lei Han, 2016 [18]	China	17	15	2	0	48 (28–72)	9.5 (4–18)	SI	RT	ORR, DCR, OS, AEs
Yanhao Liu, 2021 [19]	China	103	87	16	0	52 (29–72)	12 (2–43)	SI	RT	ORR, DCR, PFS, OS, AEs
Lina Tong, 2017 [20]	China	33	29	0	4	50.9 (25–76)	Median 16	SI	RT	ORR, DCR, PFS, OS, AEs
Ang Qu, 2018 [21]	China	36	33	3	0	44 (30–79)	11.5 (2–30)	SI	RT	ORR, DCR, PFS, OS, AEs

Abbreviations: ADCC, adenocarcinoma; AEs, adverse events; DCR, disease control rate; NR, not reported; ORR, objective response rate; OS, overall survival; PFS, progression‐free survival; RT, radiotherapy; SCC, squamous cell carcinoma; SI, seed implantation.

### 2.4 Quality Evaluation

The JBI case series key assessment checklist [24] was used for evaluating the included studies.

### 2.5 Statistical Analysis

We used STATA 16 software to analyze all of the data in this study. The *I* ^2^ statistic was employed to estimate heterogeneity. *p* < 0.1 indicates a statistically significant difference. If there was significant heterogeneity (*p* < 0.1 and *I* ^2^ > 50%), a random‐effect model was used. Otherwise, a fixed‐effect model was used. In addition, a sensitivity analysis was performed to evaluate the stability and dependability of the pooled data.

## 3 Results

### 3.1 Study Selection

**FIGURE 1:**
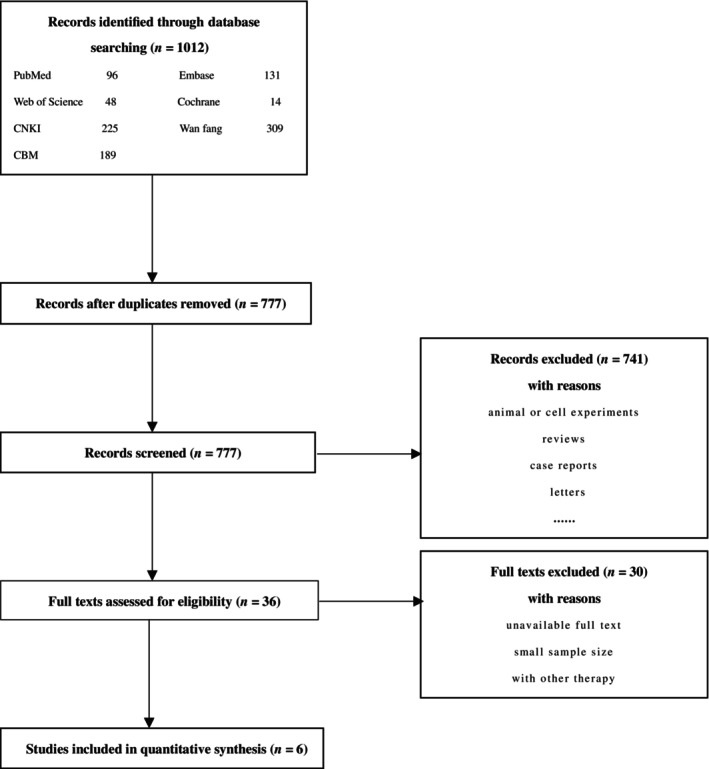
Flow chart of meta‐analysis of inclusion/exclusion studies.

A total of 1012 relevant studies were obtained from seven databases (CNKI = 225, Wanfang = 309, CBM = 189, PubMed = 96, Embase = 131, Cochrane Library = 14, Web of Science = 48). There are 777 studies left after removing duplicates. Then, according to the exclusion criteria, 741 studies were excluded, and 36 studies were retained. We assessed the remaining studies carefully, and 30 studies were eliminated because the complete text was not available, the sample size was tiny; and ^125^I seed implantation was combined with other therapies. Finally, six studies were added to this meta‐analysis since they met the inclusion criteria, and all of which were retrospective investigations. Figure 1 depicts the flowchart for the selection procedure.

### 3.2 Quality Assessment

The JBI critical appraisal checklist for case series was used to evaluate all studies. Details of the quality assessment are displayed in Table 2.

**TABLE 2:** Evaluation of the quality of studies included in meta‐analysis.

Study, year	Q1	Q2	Q3	Q4	Q5	Q6	Q7	Q8	Q9	Q10	Total
Liqiu Ji, 2021 [16]	0	2	2	2	2	0	2	2	2	2	16
Shuqin Chen, 2020 [17]	0	2	2	2	2	0	2	2	2	2	16
Lei Han, 2016 [18]	0	2	2	2	2	0	2	2	2	2	16
Yanhao Liu, 2021 [19]	2	2	2	2	2	2	2	2	2	2	20
Lina Tong, 2017 [20]	2	2	2	2	2	2	2	2	2	2	20
Ang Qu, 2018 [21]	0	2	2	2	2	2	2	2	2	2	18

*Note:* The heading's numbers Q1–Q10 indicated: Q1, were the requirements for inclusion in the case series well‐defined? Q2, was the condition assessed for each participant in the case series using a consistent, trustworthy method? Q3, were appropriate techniques employed to determine the condition of each participant in the case series? Q4, was there a sequential participation in the case series? Q5, was the case series completely representative of the participants? Q6, was the demographic information for the study participants clearly reported? Q7, was the participants' clinical information clearly reported? Q8, were there any reported case outcomes or follow‐up results? Q9, was demographic information about the presenting site(s) or clinic(s) clearly reported? Q10, was statistical analysis proper?

### 3.3 Tumor Control

**FIGURE 2:**
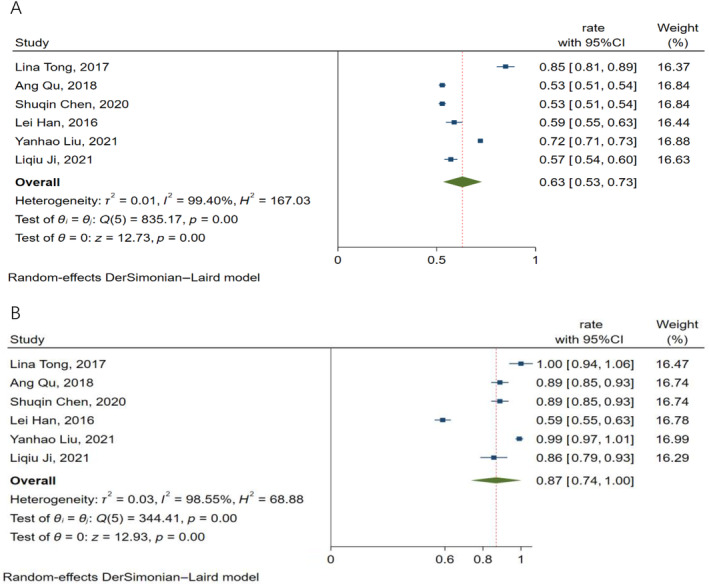
Forest plot of the pooled results of ORR (A) and DCR (B). DCR, disease control rate; ORR, objective response rate.

All studies included in the meta‐analysis reported the efficacy of ^125^I seed implantation in the treatment of pelvic recurrent cervical cancer following radiotherapy. The ORR of the included studies ranged from 53% to 85%. The use of the random‐effect model (*I* ^2^ = 99.4%, *p* < 0.01) was made because of the considerable heterogeneity. The pooled ORR was 63% (95%CI: 53%–73%). All studies also included available DCR data with significant heterogeneity (*I* ^2^ = 98.55%, *p* < 0.01) and a pooled DCR of 87% (95%CI: 74%–100%). Figure 2 depicts the forest plot of ORR and DCR.

### 3.4 Survival

**FIGURE 3:**
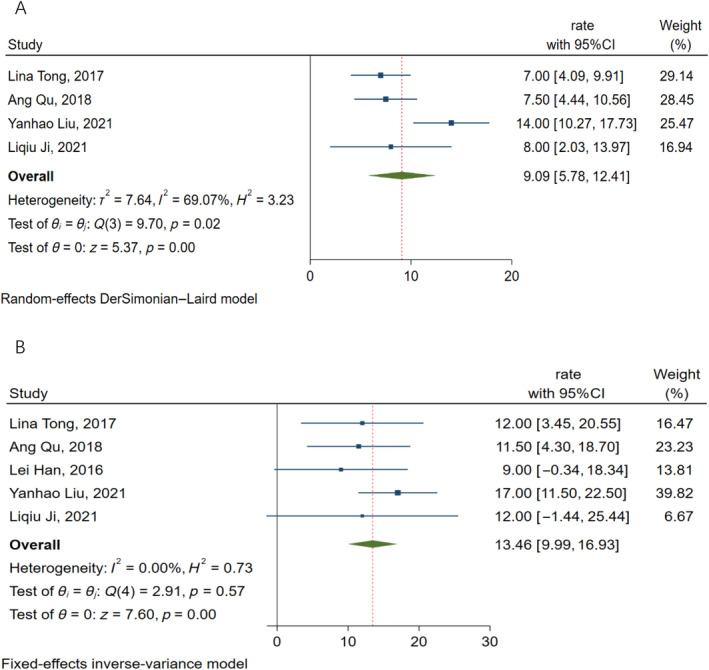
Forest plot of the pooled results of PFS (A) and OS (B). OS, overall survival; PFS, progression‐free survival.

A random‐effects model (*I* ^2^ = 69.07%, *p* < 0.01) was used to pool the median PFS, which was reported in four of the six included studies and was 9.09 months (95%CI: 5.78–12.41 months). A fixed‐effect model (*I* ^2^ = 0, *p* < 0.0 1) was used to pool the median OS, which was reported in five of the six included studies, and a meta‐analysis revealed a pooled OS of 13.46 months (95%CI: 9.99–16.93 months). Figure 3 depicts the forest plot of PFS and OS.

### 3.5 Toxicity

The most frequent adverse events (all grades and Grade ≥III) associated with ^125^I seed implantation in the treatment of pelvic recurrent cervical cancer following radiotherapy were analyzed. The majority of patients had mild to moderate adverse events. The overall incidence of toxicities was 40% (95%CI: 31%–50%). For these, the most common adverse event was proctitis, with a rate of 12% (95%CI: 8%–16%), followed by seed migration at 11% (95%CI: 7%–15%). The incidence of post procedural fever was 6% (95%CI: 0%–11%), post procedural pain aggravation was 6% (95%CI: 3%–8%), and urinary system reactions (such as frequent urination, urgent urine, etc.) was 6% (95%CI: 3%–8%). Other adverse events mentioned in the study, such as discomfort at the puncture site, local numbness, sciatic nerve injury, track metastasis, skin injury, vaginal hemorrhage, and a decrease in secretions, were reported in only one study and were not further pooled. The incidence of Grade ≥III adverse events was significantly lower, with a pooled incidence of 6% (95%CI: 6%–7%). Three of the studies included in the analysis reported the occurrence of vaginal fistula with a pooled incidence of 5% (95%CI: 4%–6%), and two of these studies explicitly mentioned the occurrence of rectovaginal fistula with an incidence of 2% (95%CI: 1%–4%). The occurrence of incomplete bowel obstruction was reported in three studies with a pooled incidence of 3% (95%CI: 3%–3%). Only one study reported the occurrence of Grade III proctitis, so no further analysis was made. Figure S1 depicts the forest plot of any grade adverse events.

### 3.6 Sensitivity Analysis

Sensitivity analysis refers to observing different models, removing low‐quality literature from the included literature according to the research quality evaluation criteria, stratifying the included study according to the sample size, changing the inclusion and exclusion criteria, re‐conducting meta‐analysis, the effect merges the differences between point estimation and interval estimation and examines whether the conclusions change or not, to ensure the robustness of meta‐analysis results. The analysis's findings show that no single study had a particularly large impact on any of the pooled outcomes with 95%CIs. This proved that the meta‐analysis's overall conclusions were generally quite trustworthy. The Supporting information displays the sensitivity analysis's findings. Figure S2 depicts the sensitivity analysis.

### 3.7 Publication Bias

A total of six studies were included in this meta‐analysis, considering that the number of included studies was less than 10, so there was no publication bias test [25].

## 4 Discussion

The most common site of recurrence following radiotherapy for cervical cancer was isolated distant recurrent metastasis (59.5%), followed by combined recurrence (central and paracervical recurrence 21.5%), then central recurrence (cervical or vaginal stump 10.5%), and lastly paracervical recurrence (pelvic lymph nodes or pelvic wall 8.3%) [26]. Both central recurrence and paracervical recurrence were pelvic recurrence. The treatment of pelvic recurrence of cervical cancer after radiotherapy is extremely difficult, which is a challenging problem. At present, cervical cancer with pelvic recurrence following radiotherapy can be treated by chemotherapy, targeted therapy, immunotherapy, surgery, and local palliative radiotherapy. The specific method should be determined according to the patient's initial treatment, the site of recurrence, the patient's age and physical status. Despite the variety of treatment methods, the therapeutic effect of recurrent cervical cancer is still not satisfactory, and the 5‐year survival rate is only about 17% [27].

Currently, the consensus of domestic experts states that the indications for ^125^I seed implantation for recurrent cervical cancer are as follows [28]: (1) Age 18–80 years old with KPS ≥80. (2) Patients who could not tolerate or refuse surgery, with pathologically confirmed recurrent cervical cancer or residual tumors (with a diameter ≤7 cm). (3) After thorough therapy, there was no systemic metastasis or stable metastases (number ≤3 lesions). (4) The expected survival is ≥3 months. (5) A suitable puncture route is available and preoperative planning is expected to meet the prescription dose requirements. (6) The patient can tolerate anesthesia and seed implantation. The ^125^I seed activity recommended by the guideline for ^125^I seed implantation in the treatment of recurrent cervical cancer is 0.4–0.5 mCi and the prescription dose is 110–130 Gy. The combined prescription dose of the six clinical studies included in this study is 104.6 Gy (78.84–130.37 Gy), which is slightly lower than the prescription dose recommended by the guideline, which may be related to the fact that the clinical research time is earlier than the guideline time, and there is no guideline to guide the treatment, and it can only be based on the clinical experience treatment is related.

According to Mabuchi [29], 52 patients with pelvic central recurrence of cervical cancer were treated with high‐dose‐rate interstitial brachytherapy (HDR‐ISBT). The results showed that the local control rate was 76.9% (40/52), the median OS was 32 months (4–156 months), and the estimated 5‐year survival rate was 52.6%. Compared with the palliative control group (23 patients with central recurrence who refused to receive HDR‐ISBT), the median OS was significantly prolonged (32 vs. 13 months, *p* < 0.0001).

Mabuchi [30] then performed radical hysterectomy in 31 patients with recurrent cervical cancer after radiotherapy, with an estimated 3‐year survival rate of 53.8%. In addition, Jurado, Alcázar, and Martinez‐Monge [31] performed pelvic dissection in 48 patients with pelvic recurrent cervical cancer following radiotherapy. Studies showed that the proportion of complete tumor resection was 65% in patients with central recurrence and 28.6% in patients with pelvic wall recurrence. The 10‐year LCR was 36.3%, 43.1% for central recurrence, and 31.5% for pelvic wall recurrence. The 10‐year disease‐specific survival (DSS) of all patients was 20.5%, 27.2% for central recurrence, and 14.9% for pelvic wall recurrence.

The ORR of a single study included in this study ranges from 53% to 85%, the pooled ORR is 63% (95%CI: 53%–73%). The DCR of a single study is 59%–99%, and the pooled DCR is 87% (95%CI: 74%–100%). The median PFS was 9.09 months (95%CI: 5.78–12.41 months) and the median OS was 13.46 months (95%CI: 9.99–16.93 months). We can see that compared with Mabuchi's [29] study, the local control rate of this study is improved (87% vs. 76.9%), indicating that the short‐term effect of seed implantation is good, but median OS has no obvious advantage (13.46 vs. 32 months), which is only slightly higher than that of the patients in the palliative treatment group (13.46 vs. 13 months). It is considered that it may be related to the late stage of the patients included in the seed implantation, the lower prescription dose of the seed implantation, the difference in the site of recurrence, the pathological type, and the poor physique of the patients who have received many rounds of chemotherapy in the past. In addition, Jurado, Alcázar, and Martinez‐Monge [31] study showed that the therapeutic effect of patients with central recurrence is better than that of patients with pelvic wall recurrence, and the follow‐up period of the study was 10 years, whereas the follow‐up period of the six clinical studies included in this study was shorter than 3 years, so it is impossible to make a comparison. However, it may suggest that we should further study the effect of recurrence site on the efficacy of seed implantation, and further prolong the follow‐up period for observing the long‐term survival. On the whole, ^125^I seed implantation is still an effective treatment for pelvic recurrent cervical cancer following radiotherapy, which can improve the short‐term efficacy and survival rate of patients.

In addition, in the six clinical studies we included, most patients experienced Grade I–II adverse events and were well tolerated. The total incidence of adverse events was 40% (95%CI: 31%–50%). Of these, the most common adverse event was proctitis with an incidence of 12% (95%CI: 8%–16%), followed by seed migration with an incidence of 11% (95%CI: 7%–15%). The incidence of Grade ≥III adverse events was significantly lower, with an incidence of 6% (95%CI: 6%–7%). The main manifestations were vaginal fistula of 5% (95%CI: 4%–6%) and incomplete intestinal obstruction of 3% (95%CI: 3%–3%). Mabuchi reported that 25% (13/52) of the patients who received HDR‐ISBT [29] experienced Grade III–IV late adverse events, of which the incidence of vaginal fistula was 17.3% (9 /52). Of the 30 patients who underwent radical hysterectomy [30], 27% (8/30) experienced serious surgical complications, of which the common late complications were fistula formation and ureteral stricture. Jurado, Alcázar, and Martinez‐Monge [31] reported that of the 48 patients who underwent pelvic dissection, the incidence of post procedural adverse events was 70.8%(34/48), with the more common adverse events being post procedural fever (23), intestinal obstruction (6), and small intestinal fistula (4). From this, we can see that the patients who underwent HDR‐ISBT, radical hysterectomy, or pelvic dissection had a high incidence of serious complications and poor quality of life. It is suggested that ^125^I seed implantation is a safe treatment for pelvic recurrent cervical cancer following radiotherapy. Therefore, considering the therapeutic efficacy and complications, ^125^I seed implantation is a safe and effective treatment.

Of course, there are some limitations to the current meta‐analysis. First, there was significant heterogeneity among the included studies. Patients are exclusively limited to those who have radiotherapy; whether they have surgery or chemotherapy is unknown. Heterogeneity may also be caused by other factors, such as histology classification and staging. Second, we only assessed the clinical efficacy and safety of the included research because they are noncontrolled trials with a limited sample size. Therefore, there is no definite conclusion. Third, we were unable to assess the efficacy of ^125^I seed implantation in the treatment of distinct tissue types of cervical cancer due to a lack of adequate pathology data on the disease. In the future, we need to further analyze the efficacy of ^125^I seed implantation in the treatment of different tissue types of cervical cancer to achieve accurate treatment. Fourth, there were very few Chinese patients in the studies included in this meta‐analysis. More research is needed to verify whether this treatment works in other populations. Therefore, the sample size should be further expanded to screen the characteristics of the population that can benefit from seed implantation therapy to maximize the efficacy of this treatment.

## 5 Conclusion

In conclusion, compared with HDR‐ISBT, radical surgery and pelvic dissection, ^125^I seed implantation has no obvious advantages in local control rate and long‐term survival in the treatment of pelvic recurrent cervical cancer following radiotherapy, but it can significantly reduce the adverse events of patients and has high safety. Compared with conventional external radiotherapy, ^125^I seed brachytherapy has several advantages. It can not only increase the therapeutic dose to tumor tissue, to improve local control rate, but also effectively reduce the radiation dose of nearby normal tissue and avoid serious complications. Therefore, considering the efficacy and adverse events of the treatment, ^125^I seed implantation is a better treatment and can be used as a remedial treatment for pelvic recurrent cervical cancer following radiotherapy to prolong the survival time of patients. However, because of the limited clinical data, more research is needed to confirm this conclusion in the future.

## Author Contributions

**Yunxin Wang:** conceptualization (equal), data curation (equal), formal analysis (equal), methodology (equal), writing – original draft (equal). **Yuhong Ma:** data curation (equal), methodology (equal), software (equal), writing – original draft (equal). **Lijuan Zou:** funding acquisition (equal), writing – original draft (equal). **Hongwei Lei:** formal analysis (equal), writing – original draft (equal). **Yun Teng:** methodology (equal), software (equal). **Fuxiu Ye:** data curation (equal), resources (equal). **Feng Zhang:** conceptualization (equal), writing – review and editing (equal). **Haichen Zhang:** conceptualization (equal), writing – review and editing (equal).

## Conflicts of Interest

The authors declare no conflicts of interest.

## Supporting information


**Figure S1.** The forest plot about the pooled results of any grade adverse events. (A) overall incidence rates of toxicities; (B) proctitis; (C) seed migration; (D) urinary system reactions (such as frequent urination, urgent urine, etc.); (E)post procedural fever; (F) post procedural pain aggravation; (G) Grade ≥III adverse events; (H) vaginal fistula; (I) rectovaginal fistula; and (J) incomplete intestinal obstruction.

**Figure S2.** Sensitivity analysis. (A) Sensitivity analysis for ORR; (B) Sensitivity analysis for DCR; (C) Sensitivity analysis for PFS; (D) Sensitivity analysis for OS; (E) Sensitivity analysis for overall incidence rates of toxicities; (F) Sensitivity analysis for proctitis; (G) Sensitivity analysis for seed migration; (H) Sensitivity analysis for urinary system reactions (such as frequent urination, urgent urine, etc.); (I) Sensitivity analysis for post procedural fever; (J) Sensitivity analysis for post procedural pain aggravation; (K) Sensitivity analysis for Grade ≥III adverse events; (L) Sensitivity analysis for vaginal fistula; (M) Sensitivity analysis for rectovaginal fistula; and (N) Sensitivity analysis for incomplete intestinal obstruction. DCR, disease control rate; ORR, objective response rate; OS, overall survival; PFS, progression‐free survival.

## Data Availability

The data that support the findings of this study are available from the corresponding author upon reasonable request.
