# Supplementary material for: Efficacy and Safety of 125I Seed Implantation in the Treatment of Pelvic Recurrent Cervical Cancer Following Radiotherapy: A Single‐Arm Meta‐Analysis of Chinese Patients
Source: Cancer Rep (Hoboken). 2024 Aug 19;7(8):e2147. doi: 10.1002/cnr2.2147 (PMC11331501; doi:10.1002/cnr2.2147)
Supplement: Supplementary file 1 — Figure S1. The forest plot about the pooled results of any grade adverse events. (A) overall incidence rates of toxicities; (B) proctitis; (C) seed migration; (D) urinary system reactions (such as frequent urination, urgent urine, etc.); (E)post procedural fever; (F) post procedural pain aggravation; (G) Grade ≥III adverse events; (H) vaginal fistula; (I) rectovaginal fistula; and (J) incomplete intestinal obstruction. [file CNR2-7-e2147-s002.pdf]

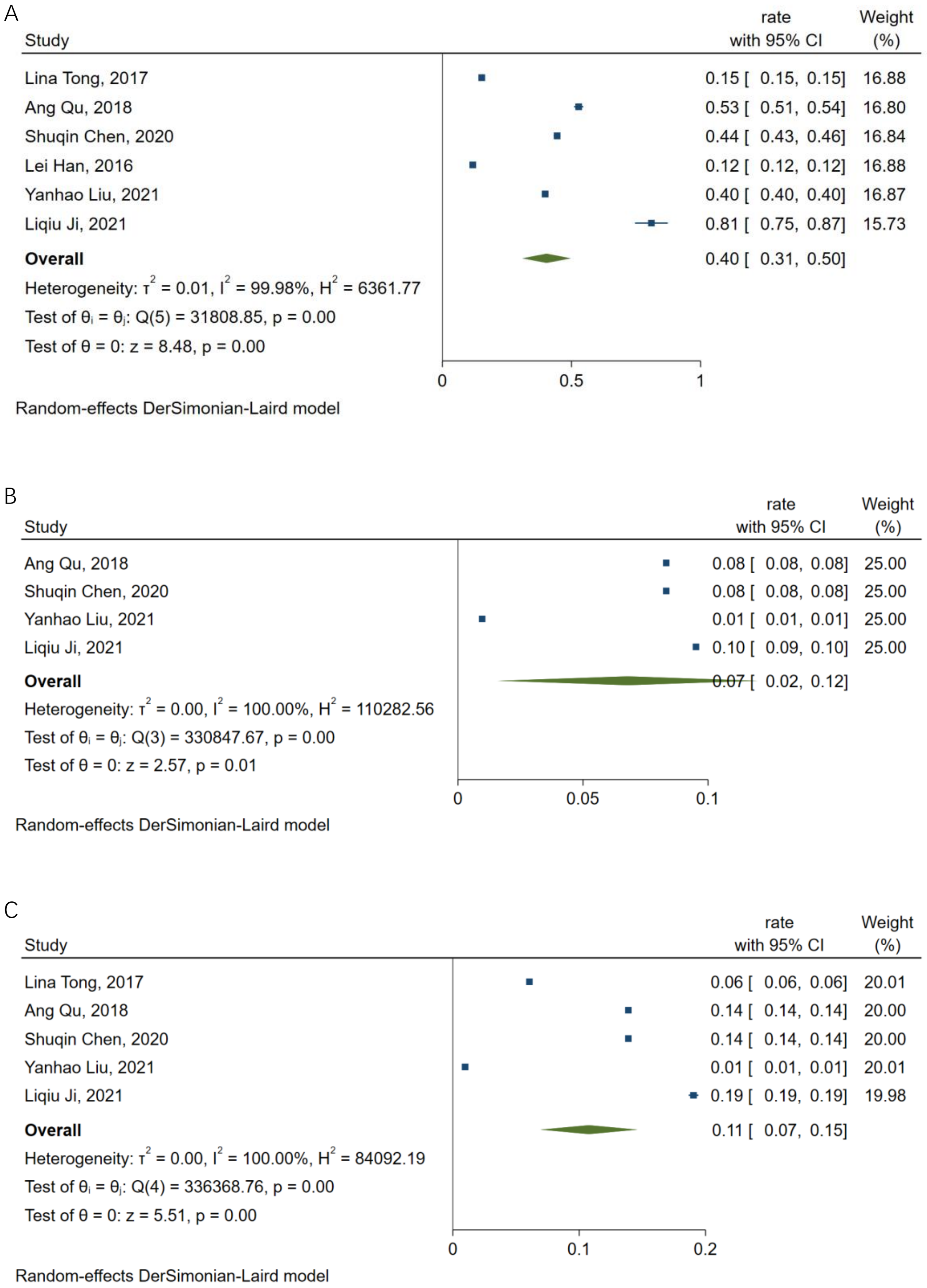
A
Study
rate with 95% CI
Weight (%)
Lina Tong, 2017 0.15 [0.15, 0.15] 16.88
Ang Qu, 2018 0.53 [0.51, 0.54] 16.80
Shuqin Chen, 2020 0.44 [0.43, 0.46] 16.84
Lei Han, 2016 0.12 [0.12, 0.12] 16.88
Yanhao Liu, 2021 0.40 [0.40, 0.40] 16.87
Liqiu Ji, 2021 0.81 [0.75, 0.87] 15.73
Overall 0.40 [0.31, 0.50]
Heterogeneity: τ² = 0.01, I² = 99.98%, H² = 6361.77
Test of θi = θj: Q(5) = 31808.85, p = 0.00
Test of θ = 0: z = 8.48, p = 0.00
0
0.5
1
Random-effects DerSimonian-Laird model
B
Study
rate with 95% CI
Weight (%)
Ang Qu, 2018 0.08 [0.08, 0.08] 25.00
Shuqin Chen, 2020 0.08 [0.08, 0.08] 25.00
Yanhao Liu, 2021 0.01 [0.01, 0.01] 25.00
Liqiu Ji, 2021 0.10 [0.09, 0.10] 25.00
Overall 0.07 [0.02, 0.12]
Heterogeneity: τ² = 0.00, I² = 100.00%, H² = 110282.56
Test of θi = θj: Q(3) = 330847.67, p = 0.00
Test of θ = 0: z = 2.57, p = 0.01
0
0.05
0.1
Random-effects DerSimonian-Laird model
C
Study
rate with 95% CI
Weight (%)
Lina Tong, 2017 0.06 [0.06, 0.06] 20.01
Ang Qu, 2018 0.14 [0.14, 0.14] 20.00
Shuqin Chen, 2020 0.14 [0.14, 0.14] 20.00
Yanhao Liu, 2021 0.01 [0.01, 0.01] 20.01
Liqiu Ji, 2021 0.19 [0.19, 0.19] 19.98
Overall 0.11 [0.07, 0.15]
Heterogeneity: τ² = 0.00, I² = 100.00%, H² = 84092.19
Test of θi = θj: Q(4) = 336368.76, p = 0.00
Test of θ = 0: z = 5.51, p = 0.00
0
0.1
0.2
Random-effects DerSimonian-Laird model

D

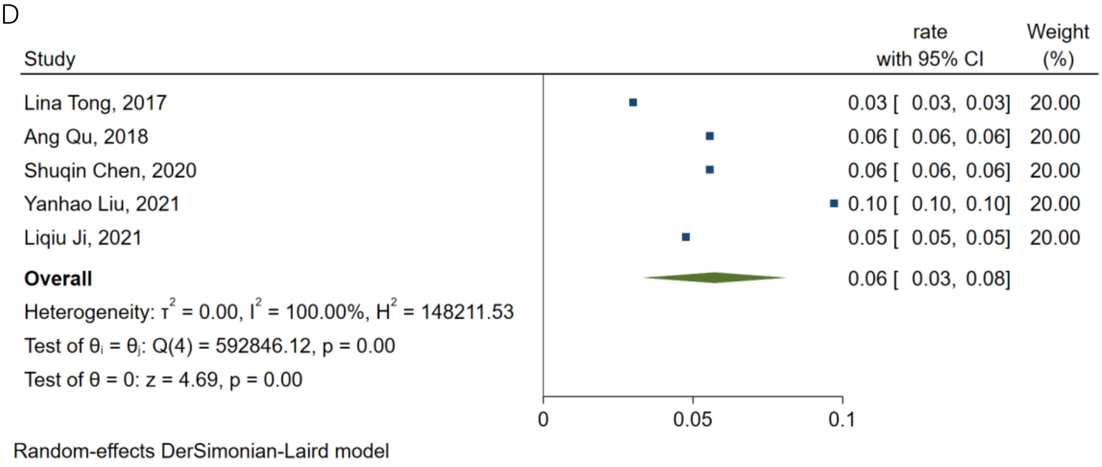
Study
rate with 95% CI
Weight (%)
Lina Tong, 2017 0.03 [0.03, 0.03] 20.00
Ang Qu, 2018 0.06 [0.06, 0.06] 20.00
Shuqin Chen, 2020 0.06 [0.06, 0.06] 20.00
Yanhao Liu, 2021 0.10 [0.10, 0.10] 20.00
Liqiu Ji, 2021 0.05 [0.05, 0.05] 20.00
Overall 0.06 [0.03, 0.08]
Heterogeneity: τ² = 0.00, I² = 100.00%, H² = 148211.53
Test of θi = θj: Q(4) = 592846.12, p = 0.00
Test of θ = 0: z = 4.69, p = 0.00
0
0.05
0.1
Random-effects DerSimonian-Laird model

E

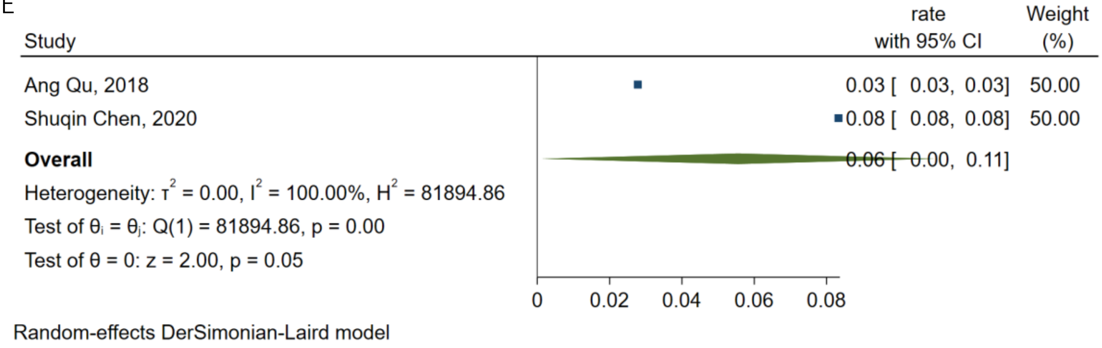
Study
rate with 95% CI
Weight (%)
Ang Qu, 2018 0.03 [0.03, 0.03] 50.00
Shuqin Chen, 2020 0.08 [0.08, 0.08] 50.00
Overall 0.06 [0.00, 0.11]
Heterogeneity: τ² = 0.00, I² = 100.00%, H² = 81894.86
Test of θi = θj: Q(1) = 81894.86, p = 0.00
Test of θ = 0: z = 2.00, p = 0.05
0
0.02
0.04
0.06
0.08
Random-effects DerSimonian-Laird model

F

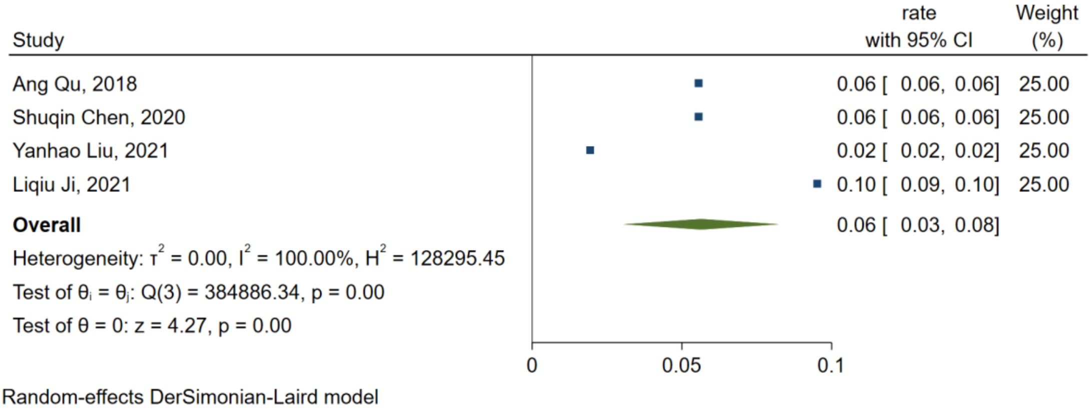
Study
rate with 95% CI
Weight (%)
Ang Qu, 2018 0.06 [0.06, 0.06] 25.00
Shuqin Chen, 2020 0.06 [0.06, 0.06] 25.00
Yanhao Liu, 2021 0.02 [0.02, 0.02] 25.00
Liqiu Ji, 2021 0.10 [0.09, 0.10] 25.00
Overall 0.06 [0.03, 0.08]
Heterogeneity: τ² = 0.00, I² = 100.00%, H² = 128295.45
Test of θi = θj: Q(3) = 384886.34, p = 0.00
Test of θ = 0: z = 4.27, p = 0.00
0
0.05
0.1
Random-effects DerSimonian-Laird model

G

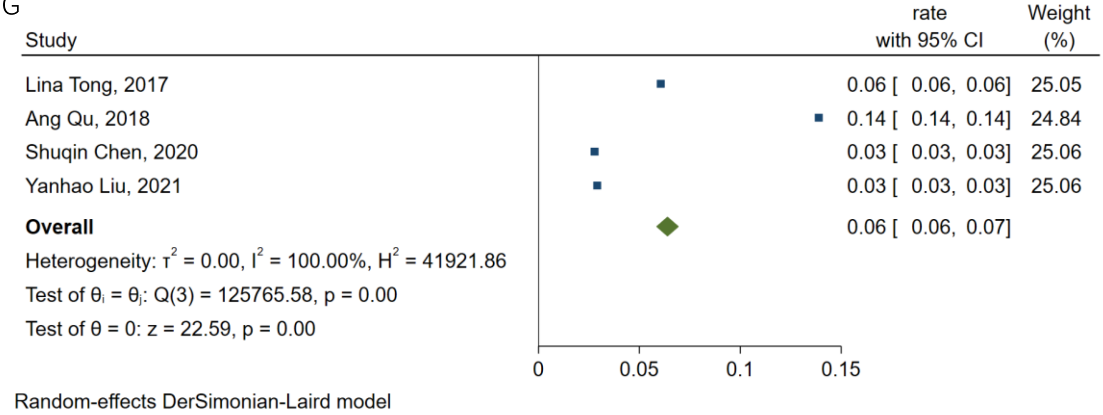
Study
rate with 95% CI
Weight (%)
Lina Tong, 2017 0.06 [0.06, 0.06] 25.05
Ang Qu, 2018 0.14 [0.14, 0.14] 24.84
Shuqin Chen, 2020 0.03 [0.03, 0.03] 25.06
Yanhao Liu, 2021 0.03 [0.03, 0.03] 25.06
Overall 0.06 [0.06, 0.07]
Heterogeneity: τ² = 0.00, I² = 100.00%, H² = 41921.86
Test of θi = θj: Q(3) = 125765.58, p = 0.00
Test of θ = 0: z = 22.59, p = 0.00
0
0.05
0.1
0.15
Random-effects DerSimonian-Laird model

H

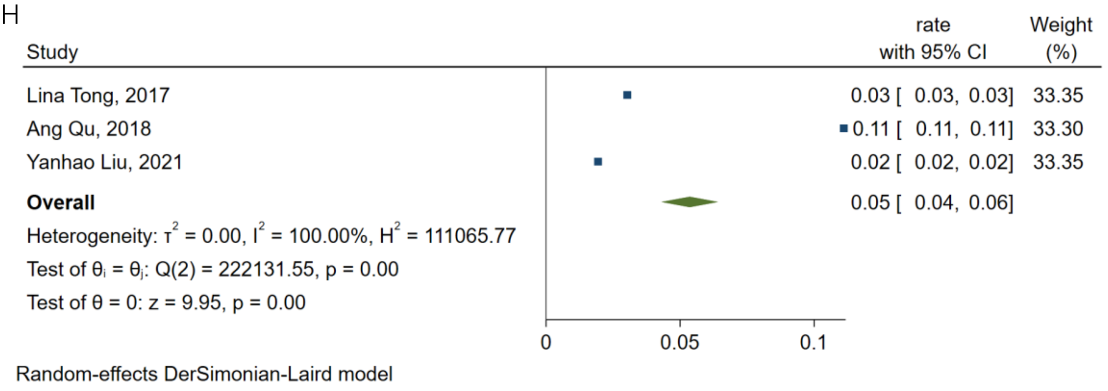

I

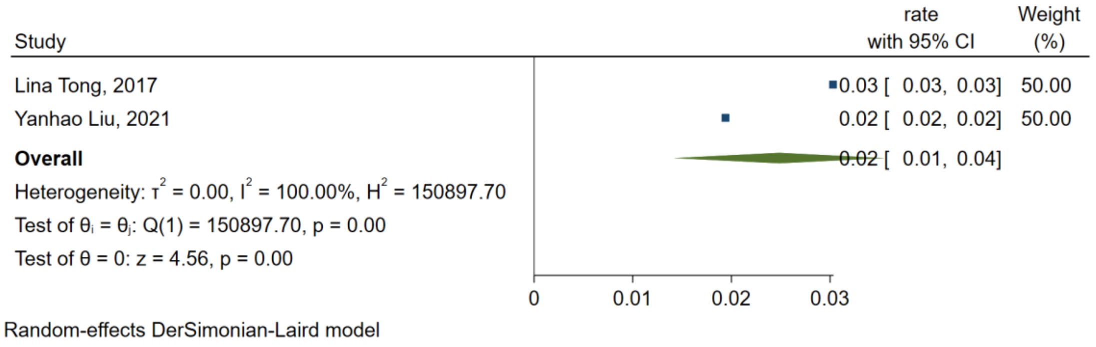

J

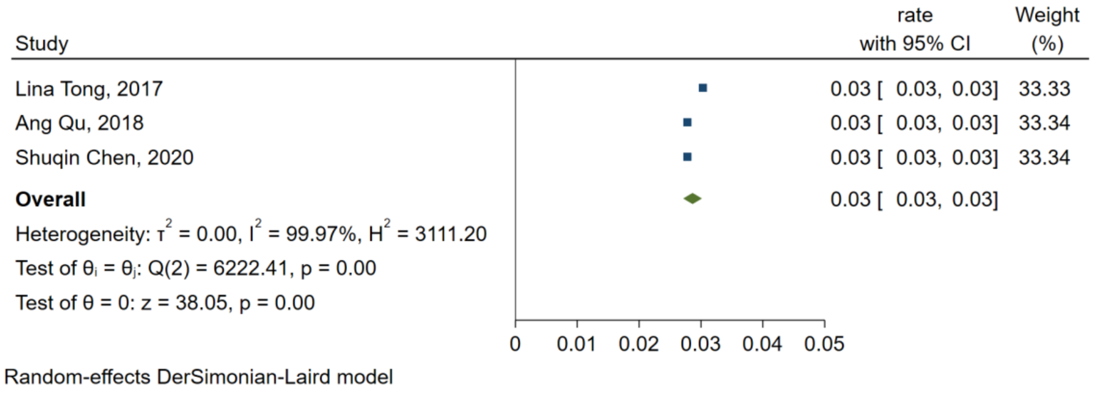

Figure S1: The forest plot about the pooled results of Any Grade adverse events. (A) overall incidence rates of toxicities; (B) proctitis; (C) seed migration; (D) urinary system reactions (such as frequent urination, urgent urine, etc.); (E)post procedural fever; (F) post procedural pain aggravation; (G)grade ≥ III adverse events; (H)vaginal fistula; (I)rectovaginal fistula; (J) incomplete intestinal obstruction.
