# Supplementary material for: Efficacy and Safety of 125I Seed Implantation in the Treatment of Pelvic Recurrent Cervical Cancer Following Radiotherapy: A Single‐Arm Meta‐Analysis of Chinese Patients
Source: Cancer Rep (Hoboken). 2024 Aug 19;7(8):e2147. doi: 10.1002/cnr2.2147 (PMC11331501; doi:10.1002/cnr2.2147)
Supplement: Supplementary file 2 — Figure S2. Sensitivity analysis. (A) Sensitivity analysis for ORR; (B) Sensitivity analysis for DCR; (C) Sensitivity analysis for PFS; (D) Sensitivity analysis for OS; (E) Sensitivity analysis for overall incidence rates of toxicities; (F) Sensitivity analysis for proctitis; (G) Sensitivity analysis for seed migration; (H) Sensitivity analysis for urinary system reactions (such as frequent urination, urgent urine, etc.); (I) Sensitivity analysis for post procedural fever; (J) Sensitivity analysis for post procedural pain aggravation; (K) Sensitivity analysis for Grade ≥III adverse events; (L) Sensitivity analysis for vaginal fistula; (M) Sensitivity analysis for rectovaginal fistula; and (N) Sensitivity analysis for incomplete intestinal obstruction. DCR, disease control rate; ORR, objective response rate; OS, overall survival; PFS, progression‐free survival. [file CNR2-7-e2147-s001.pdf]

A

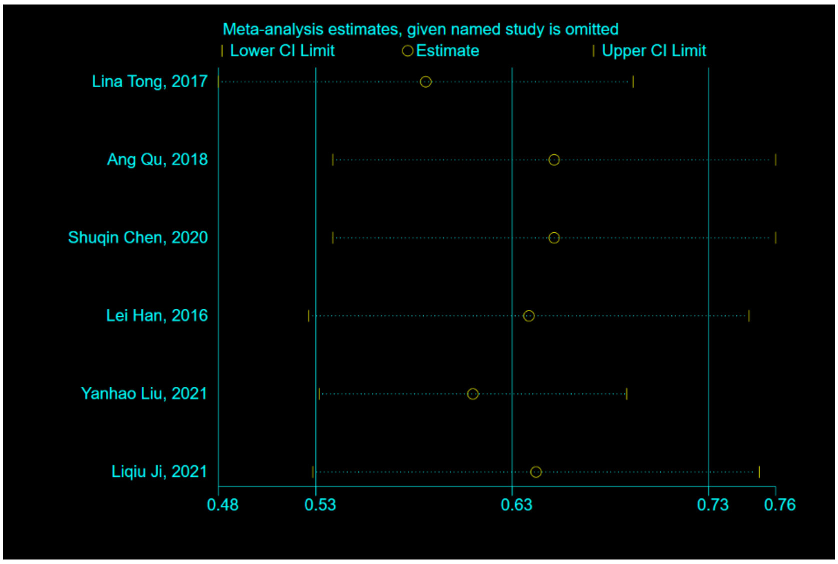

Meta-analysis estimates, given named study is omitted
Lower CI Limit
Estimate
Upper CI Limit
Lina Tong, 2017
Ang Qu, 2018
Shuqin Chen, 2020
Lei Han, 2016
Yanhao Liu, 2021
Liqiu Ji, 2021
0.48
0.53
0.63
0.73
0.76

B

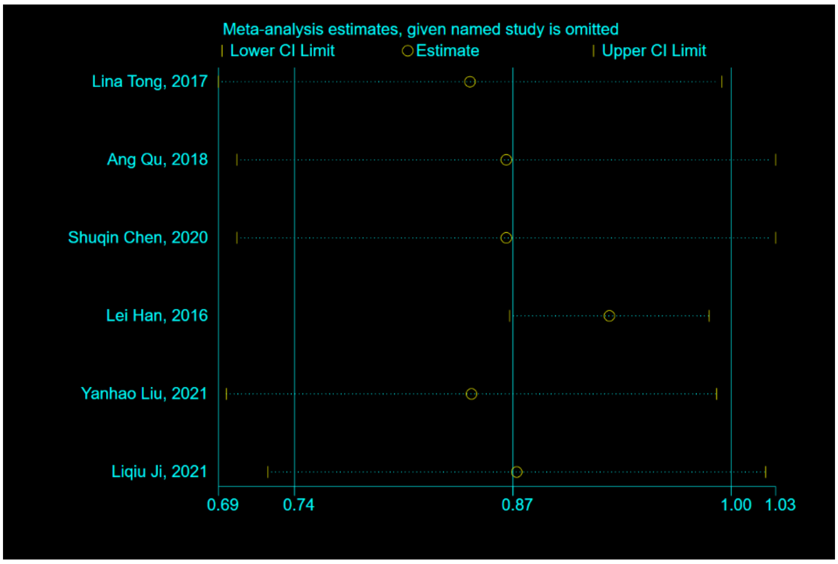

Meta-analysis estimates, given named study is omitted
Lower CI Limit
Estimate
Upper CI Limit
Lina Tong, 2017
Ang Qu, 2018
Shuqin Chen, 2020
Lei Han, 2016
Yanhao Liu, 2021
Liqiu Ji, 2021
0.69
0.74
0.87
1.00
1.03

C

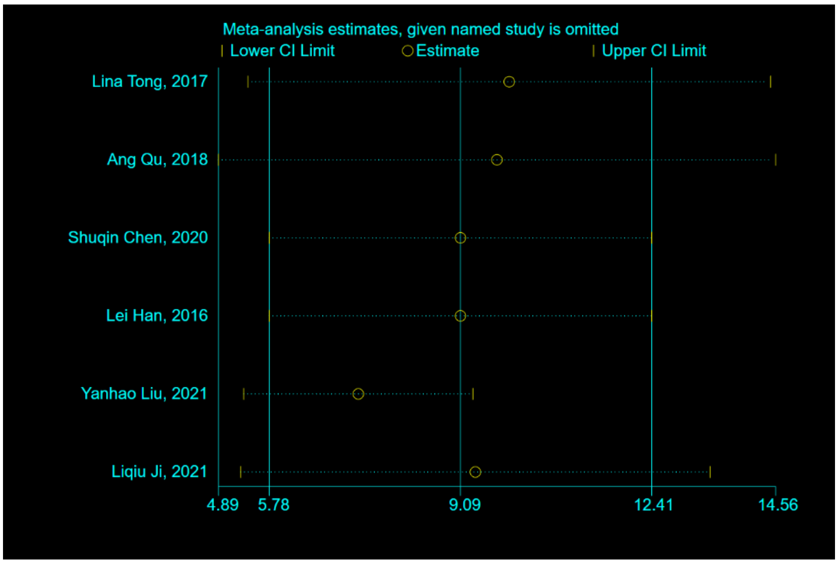

Meta-analysis estimates, given named study is omitted
Lower CI Limit
Estimate
Upper CI Limit
Lina Tong, 2017
Ang Qu, 2018
Shuqin Chen, 2020
Lei Han, 2016
Yanhao Liu, 2021
Liqiu Ji, 2021
4.89
5.78
9.09
12.41
14.56

D

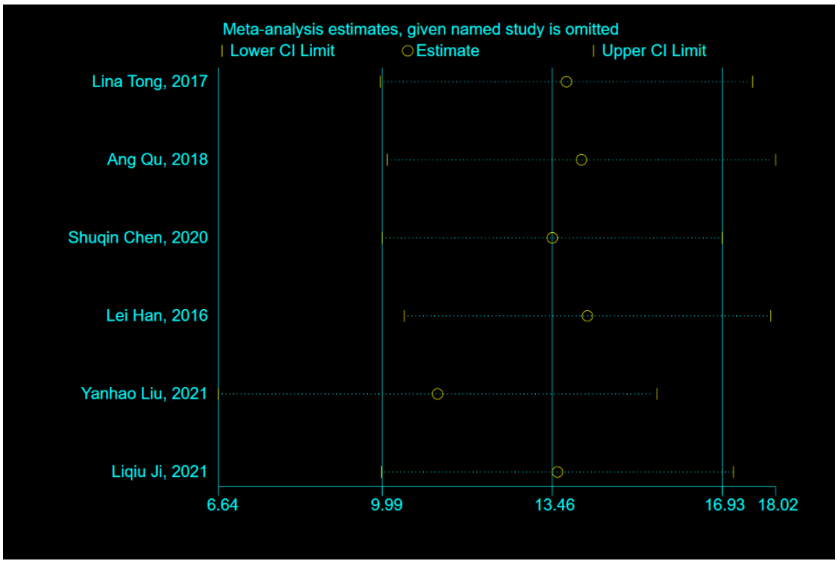

Meta-analysis estimates, given named study is omitted
Lower CI Limit
Estimate
Upper CI Limit
Lina Tong, 2017
Ang Qu, 2018
Shuqin Chen, 2020
Lei Han, 2016
Yanhao Liu, 2021
Liqiu Ji, 2021
6.64
9.99
13.46
16.93
18.02

E

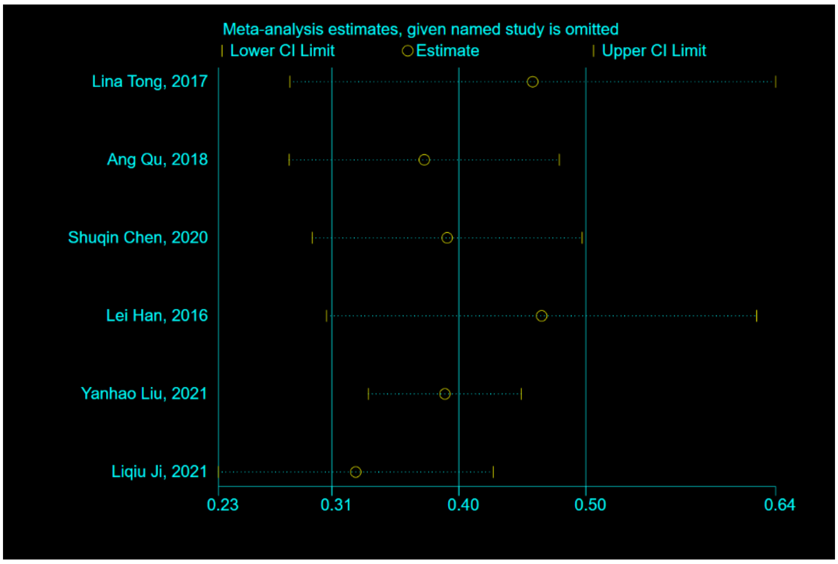

Meta-analysis estimates, given named study is omitted
Lower CI Limit
Estimate
Upper CI Limit
Lina Tong, 2017
Ang Qu, 2018
Shuqin Chen, 2020
Lei Han, 2016
Yanhao Liu, 2021
Liqiu Ji, 2021
0.23
0.31
0.40
0.50
0.64

F

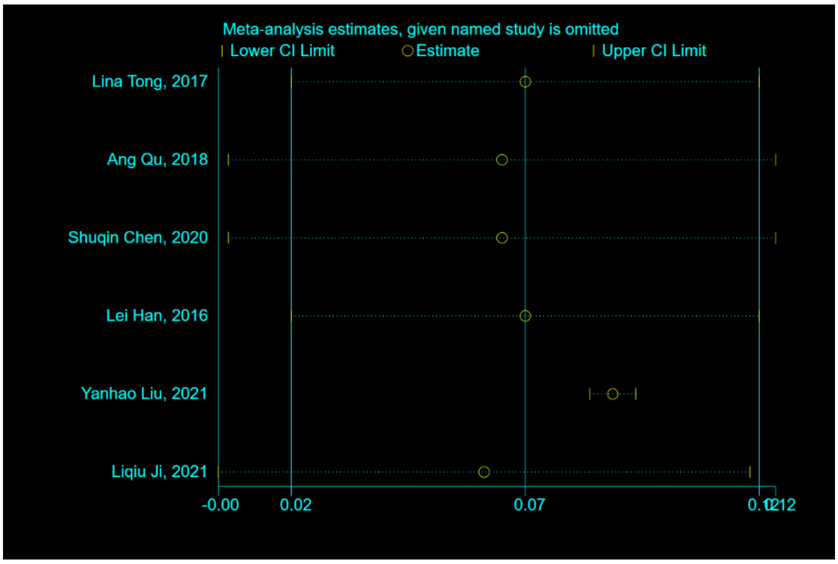

Meta-analysis estimates, given named study is omitted
Lower CI Limit
Estimate
Upper CI Limit
Lina Tong, 2017
Ang Qu, 2018
Shuqin Chen, 2020
Lei Han, 2016
Yanhao Liu, 2021
Liqiu Ji, 2021
-0.00
0.02
0.07

G

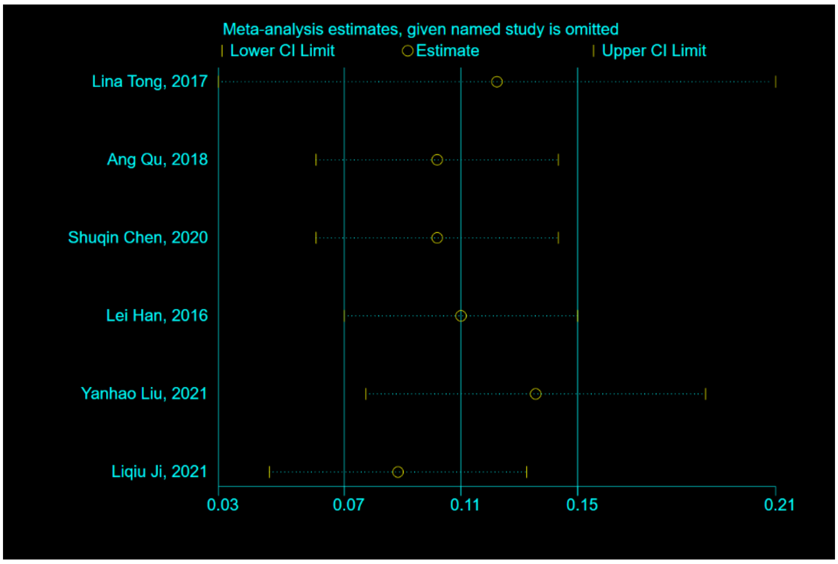

Meta-analysis estimates, given named study is omitted
Lower CI Limit
Estimate
Upper CI Limit
Lina Tong, 2017
Ang Qu, 2018
Shuqin Chen, 2020
Lei Han, 2016
Yanhao Liu, 2021
Liqiu Ji, 2021
0.03
0.07
0.11
0.15
0.21

H

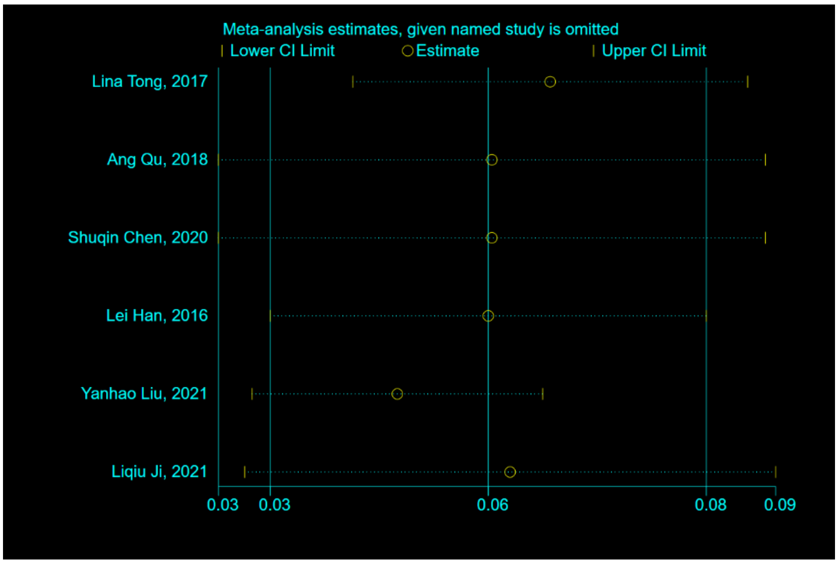

Meta-analysis estimates, given named study is omitted
Lower CI Limit
Estimate
Upper CI Limit
Lina Tong, 2017
Ang Qu, 2018
Shuqin Chen, 2020
Lei Han, 2016
Yanhao Liu, 2021
Liqiu Ji, 2021
0.03
0.03
0.06
0.08
0.09

I

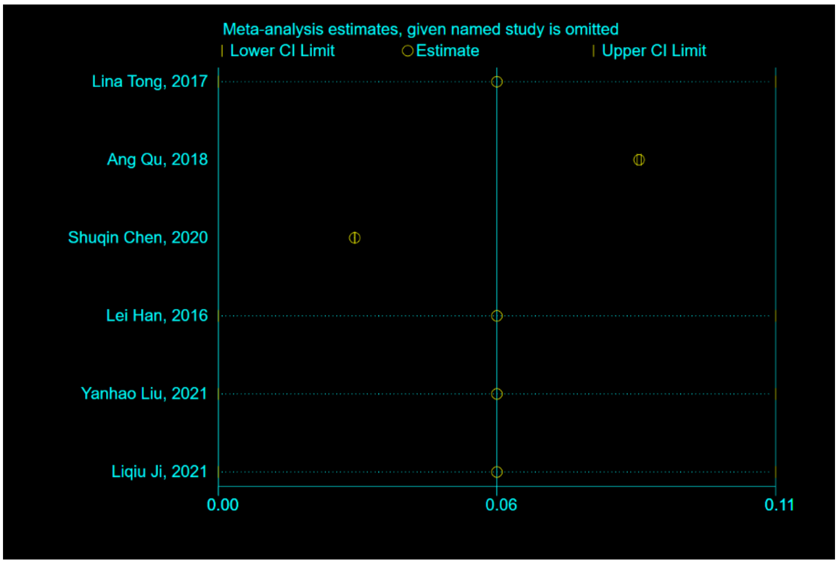

Meta-analysis estimates, given named study is omitted
Lower CI Limit
Estimate
Upper CI Limit
Lina Tong, 2017
Ang Qu, 2018
Shuqin Chen, 2020
Lei Han, 2016
Yanhao Liu, 2021
Liqiu Ji, 2021
0.00
0.06
0.11

J

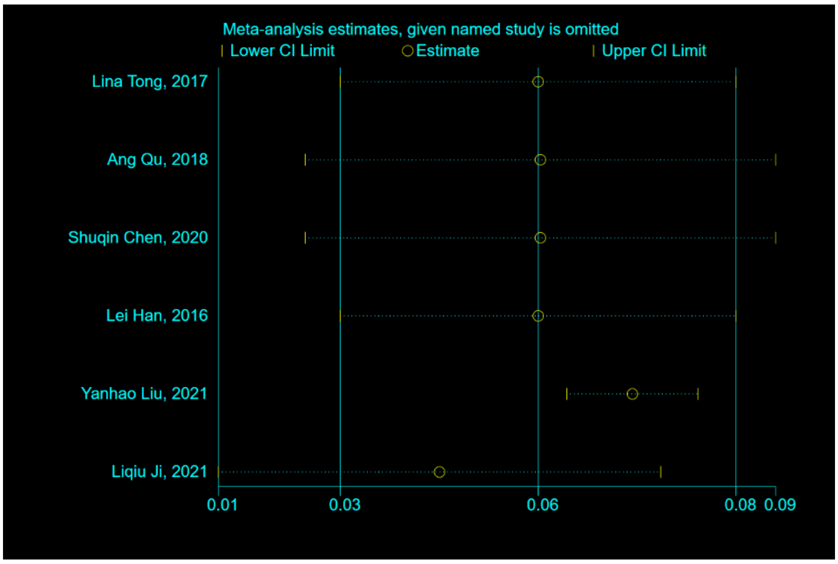

Meta-analysis estimates, given named study is omitted
Lower CI Limit
Estimate
Upper CI Limit
Lina Tong, 2017
Ang Qu, 2018
Shuqin Chen, 2020
Lei Han, 2016
Yanhao Liu, 2021
Liqiu Ji, 2021
0.01
0.03
0.06
0.08
0.09

K

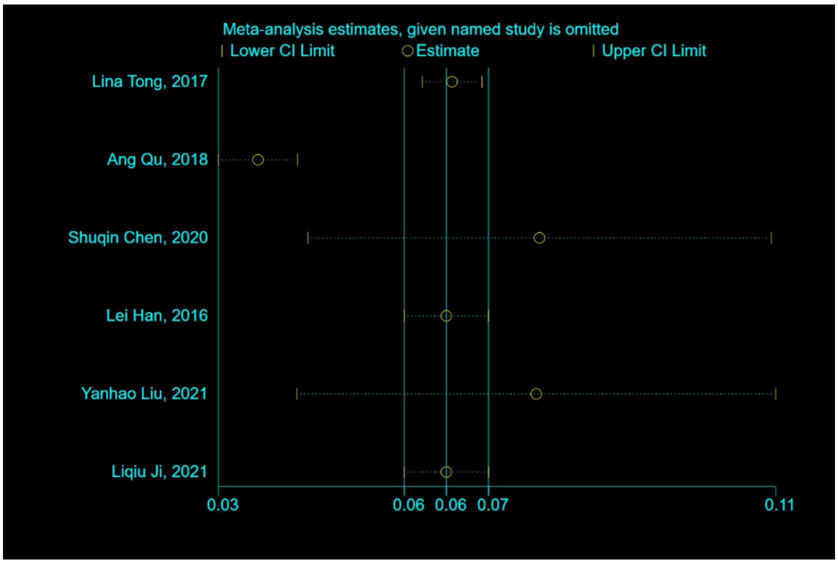

Meta-analysis estimates, given named study is omitted
Lower CI Limit
Estimate
Upper CI Limit
Lina Tong, 2017
Ang Qu, 2018
Shuqin Chen, 2020
Lei Han, 2016
Yanhao Liu, 2021
Liqiu Ji, 2021
0.03
0.06
0.06
0.07
0.11

L

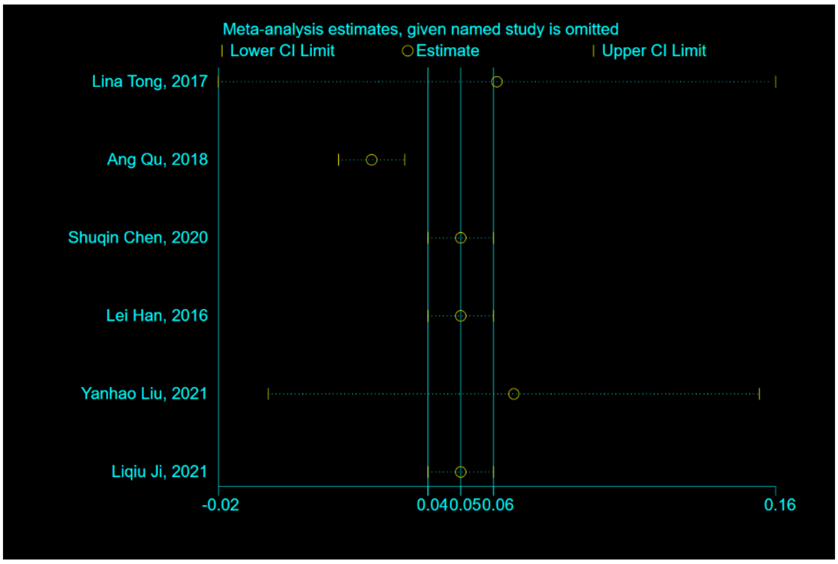

Meta-analysis estimates, given named study is omitted
Lower CI Limit
Estimate
Upper CI Limit
Lina Tong, 2017
Ang Qu, 2018
Shuqin Chen, 2020
Lei Han, 2016
Yanhao Liu, 2021
Liqiu Ji, 2021
-0.02
0.040.050.06
0.16

M

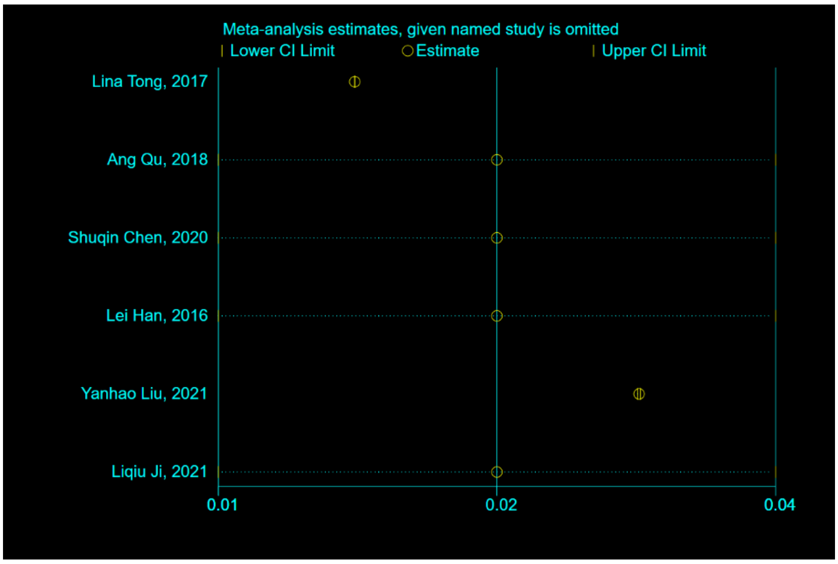

N

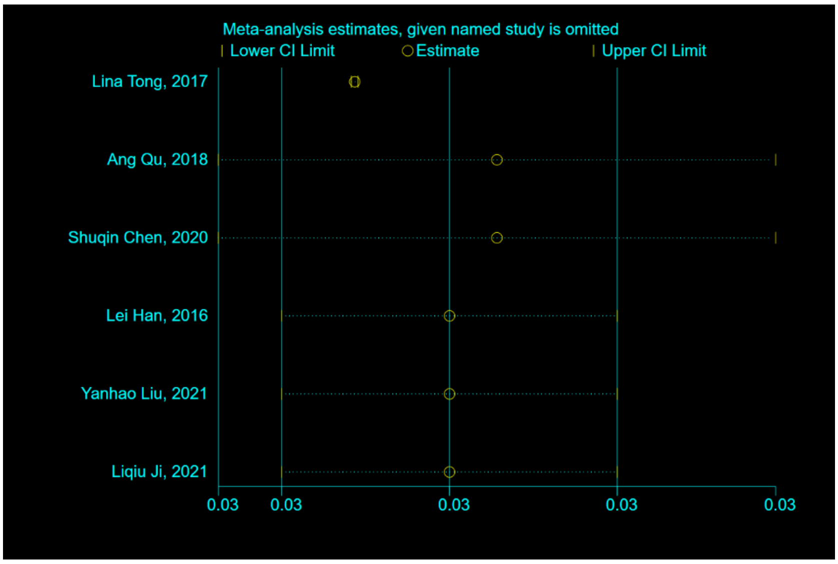

Figure S2: Sensitivity analysis. (A) Sensitivity analysis for ORR; (B) Sensitivity analysis for DCR; (C) Sensitivity analysis for PFS; (D) Sensitivity analysis for OS; (E) Sensitivity analysis for overall incidence rates of toxicities; (F) Sensitivity analysis for proctitis; (G) Sensitivity analysis for seed migration; (H) Sensitivity analysis for urinary system reactions (such as frequent urination, urgent urine, etc.); (I) Sensitivity analysis for post procedural fever; (J) Sensitivity analysis for post procedural pain aggravation; (K) Sensitivity analysis for grade ≥ III adverse events; (L) Sensitivity analysis for vaginal fistula; (M) Sensitivity analysis for rectovaginal fistula; (N) Sensitivity analysis for incomplete intestinal obstruction. ORR, objective response rate; DCR, disease control rate; PFS, progression-free survival; OS, overall survival.
